# Heat Killed *Lactobacillus reuteri* GMNL-263 Reduces Fibrosis Effects on the Liver and Heart in High Fat Diet-Hamsters via TGF-β Suppression

**DOI:** 10.3390/ijms161025881

**Published:** 2015-10-28

**Authors:** Wei-Jen Ting, Wei-Wen Kuo, Dennis Jine-Yuan Hsieh, Yu-Lan Yeh, Cecilia-Hsuan Day, Ya-Hui Chen, Ray-Jade Chen, Viswanadha Vijaya Padma, Yi-Hsing Chen, Chih-Yang Huang

**Affiliations:** 1 Graduate Institute of Basic Medical Science, China Medical University, Taichung 40402, Taiwan; E-Mail: Wei-Jen.Ting@outlook.com; 2 Department of Biological Science and Technology, China Medical University, Taichung 40402, Taiwan; E-Mail: wwkuo@mail.cmu.edu.tw; 3 School of Medical Laboratory and Biotechnology, Chung Shan Medical University, Taichung 40201, Taiwan; E-Mail: djh@csmu.edu.tw; 4 Department of Pathology, Changhua Christian Hospital, Changhua 50006, Taiwan; E-Mail: 1867@cch.org.tw; 5 Jen-Teh Junior College of Medicine, Nursing and Management, Miaoli 35664, Taiwan; 6 Department of Nursing, Mei Ho University, Pingtung 91201, Taiwan; E-Mail: x00003023@meiho.edu.tw; 7 Research and Development Department, GenMont Biotech Incorporation, Tainan 74144, Taiwan; E-Mail: yahui@genmont.com.tw; 8 Department of Surgery, School of Medicine, College of Medicine, Taipei Medical University, Taipei 11031, Taiwan; E-Mail: rayjchen@tmu.edu.tw; 9 Department of Biotechnology, Bharathiar University, Coimbatore 641046, India; E-Mail: padama.vijaya@gmail.com; 10 Graduate Institute of Chinese Medical Science, China Medical University, Taichung 40402, Taiwan; 11 Department of Health and Nutrition Biotechnology, Asia University, Taichung 41354, Taiwan

**Keywords:** *Lactobacillus reuteri* GMNL-263, transforming growth factor β, cardiovascular diseases

## Abstract

Obesity is one of the major risk factors for nonalcoholic fatty liver disease (NAFLD), and NAFLD is highly associated with an increased risk of cardiovascular disease (CVD). Scholars have suggested that certain probiotics may significantly impact cardiovascular health, particularly certain Lactobacillus species, such as *Lactobacillus reuteri* GMNL-263 (Lr263) probiotics, which have been shown to reduce obesity and arteriosclerosis *in vivo*. In the present study, we examined the potential of heat-killed bacteria to attenuate high fat diet (HFD)-induced hepatic and cardiac damages and the possible underlying mechanism of the positive effects of heat-killed Lr263 oral supplements. Heat-killed Lr263 treatments (625 and 3125 mg/kg-hamster/day) were provided as a daily supplement by oral gavage to HFD-fed hamsters for eight weeks. The results show that heat-killed Lr263 treatments reduce fatty liver syndrome. Moreover, heat-killed *Lactobacillus reuteri* GMNL-263 supplementation in HFD hamsters also reduced fibrosis in the liver and heart by reducing transforming growth factor β (TGF-β) expression levels. In conclusion, heat-killed Lr263 can reduce lipid metabolic stress in HFD hamsters and decrease the risk of fatty liver and cardiovascular disease.

## 1. Introduction

Metabolic syndrome comprises of hypertension, dyslipidemia, obesity, glucose intolerance, and cardiovascular disease (CVD) [1,2,3,4,5]. Lipid metabolic disorders and an increase in adipose tissue accompany cardiovascular disease, particularly with obesity [6]. The main cause of obesity is excessive calorie and sugar intake [7]. An investigation into the relationship between general and central obesity revealed that all-cause and CVD-related mortality in an Asian population exhibited higher central-obesity indices, such as waist circumference (WC) [8].

In previous studies, carbon tetrachloride-induced liver injury in animal experiments showed that liver damage-induced abnormal lipid metabolism increased both cholesterol and transforming growth factor β (TGF-β) levels in blood [9,10]. Overexpression of TGF-β from a damaged liver may cause cirrhotic cardiomyopathy (CCM) [11,12]. Furthermore, lipid metabolism abnormalities in the liver also cause coagulation function disorders because many clotting factors are synthesized and secreted by the liver [13,14].

The TGF-β cytokines are pleiotropic and implicated in a wide variety of extra-cellular matrix deposition, cell proliferation, and differentiation pathways [15]. Connective-tissue growth factor (CTGF) and endothelin may also be induced and expressed as TGF-β downstream effectors [16]. TGF-β-induced CTGF expression can lead to cardiomyocyte hypertrophy and fibroblast proliferation; these changes contribute to cardiac remodeling [17,18].

Recently, certain reports have revealed the effects of certain probiotic strains on cholesterol and hypertension reduction, and these data suggest that probiotics could be more widely applied for cardiovascular health [19,20,21]. Reports show that *Lactobacillus reuteri* is a probiotic species with a serum cholesterol-lowering ability in humans [22]. The most accepted mechanism underlying these effects is that *Lactobacillus* features bile salt hydrolase (BSH) activity, which suggests that probiotics may cause deconjugation effects in primary bile acids and promote the secondary bile salts by amino acid conjugations in the gut [23]. These effects will break down the cholesterol-bile salt reabsorption and lower the cholesterol levels of the hosts [24,25,26].

Cholesterol and hypertension are risk factors associated with obesity in causing heart disease; these risks are reduced by nearly half when cholesterol and hypertension decrease [19]. Recently, two reports showed that oral *Lactobacillus reuteri* GMNL-263 (Lr263) administration can prevent renal fibrosis in a diabetic kidney, improve insulin resistance, and ameliorate hepatic steatosis in high fructose-fed rats [27,28]. However, probiotic administration may cause significant change to the gut biota profile in the host. Therefore heat-killed bacteria have become an attractive future strategy to simulate the effects of probiotics. Previous studies show that heat-killed Lr263 potentially improved in heart function against the effects of HFD [29]. In our previous research, a high-fat diet treatment caused obesity and cardiac fibrosis in an animal model [21,26,28]. In this work, a high-fat diet was employed to induce obesity and cardiac fibrosis in hamsters; the protective effects exerted by different doses of heat-killed Lr263 on the heart and liver were also investigated in hamsters with high-fat diet-induced obesity.

## 2. Results and Discussion

**Figure 1:**
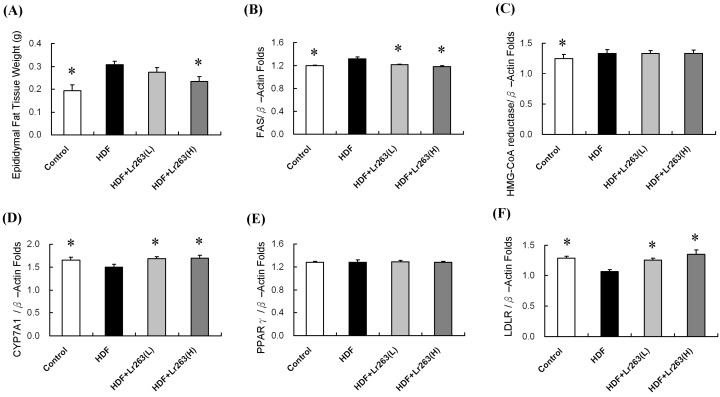
The animal epididymal adipose tissue weight and liver lipid and cholesterol metabolism biomarkers. (**A**) The HFD hamster epididymal adipose tissue weights were higher than in the control group, and the apididymal adipose tissue weight was lower in the heat-killed Lr263 (625 and 3125 mg/kg-hamster/day) treatment groups; (**B**) FAS; (**C**) HMG-CoA reductase; (**D**) CYP7A1; (**E**) PPARγ; and (**F**) LDLR are liver lipid metabolism biomarkers and were analyzed using RT-PCR. FAS and HMG-CoA reductase were greater in the HFD group and lower in the heat killed Lr263 (3125 mg/kg-hamster/day) treatment groups, but the difference in HMG-CoA reductase was not significant in the heat-killed Lr263 treatment groups. LDLR and CYP7A1 was lower in the HFD group and greater in the heat killed Lr263 (625 and 3125 mg/kg-hamster/day) treatment groups. (* *p* < 0.05 compared with the HFD group).

Epididymal adipose tissue comprises the body fat tissue of an animal [30]. After two months of experimentation, the epididymal adipose tissue mass in the HFD-only hamsters was greater than in the controls (Figure 1A). The epididymal adipose mass decreased in a dose-dependent manner for the heat-killed Lr263 (625 and 3125 mg/kg-hamster/day) treatment groups. Furthermore, fatty acid synthase (FAS) is a biomarker of liver lipid metabolism [31,32]. After the RT-PCR analysis, FAS increased in the HFD-only group and decreased in the heat-killed Lr263 (625 and 3125 mg/kg-hamster/day) treatment groups (Figure 1B). However, HMG-CoA reductase increased in the HFD group rats and decreased only slightly in the heat-killed Lr263 treatment groups, which was not significant (Figure 1C). LDLR and CYP7A1 are liver cholesterol metabolism biomarkers [33]. After the RT-PCR analysis, LDLR and CYP7A1 were lower in the HFD-only group and greater in the heat-killed Lr263 (625 and 3125 mg/kg-hamster/day) treatment groups (Figure 1D–F).

Nonalcoholic fatty liver disease (NAFLD) is associated with an increased risk of cirrhosis in both obese adults and children [34]. Certain studies have shown that the fibrosis severity stage in non-alcoholic fatty liver disease (NAFLD) patients was highly associated with mortality from cardiovascular causes and cardiac risk [35,36,37]. Supplementation with probiotics to reset the symbiotic obese gut microbiome may be an approach to improving outcomes [19].

One study found no drastic change in food intake in *Lactobacillus rhamnosus* GG treated mice when compared to control C57BL mice, and the significant weight decrease in epididymal fat tissue was not because of reduced energy intake [38]. This experimental result suggests an anti-obesity effect of *Lactobacillus rhamnosus* GG administration is directly through epididymal fat mass reduction. [38]. In this work, heat killed Lr263 oral gavage treatment was provided for eight weeks and exhibited a similar effect, directly decreasing epididymal fat mass in HFD hamsters (Figure 1A). Furthermore, lipid and cholesterol metabolic function in the HFD hamster liver improved with heat-killed Lr263 treatments in a dose-dependent manner (Figure 1).

**Figure 2:**
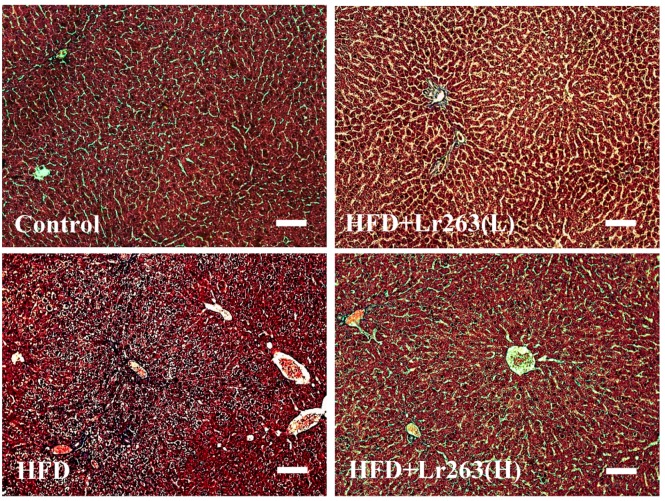
Fibrosis assessments. Cirrhosis was assessed using Massion’s trichrome (MS) staining assay to indicate the collagen accumulation (by the blue color) in liver tissue slides. The bar length is 100 μm.

**Figure 3:**
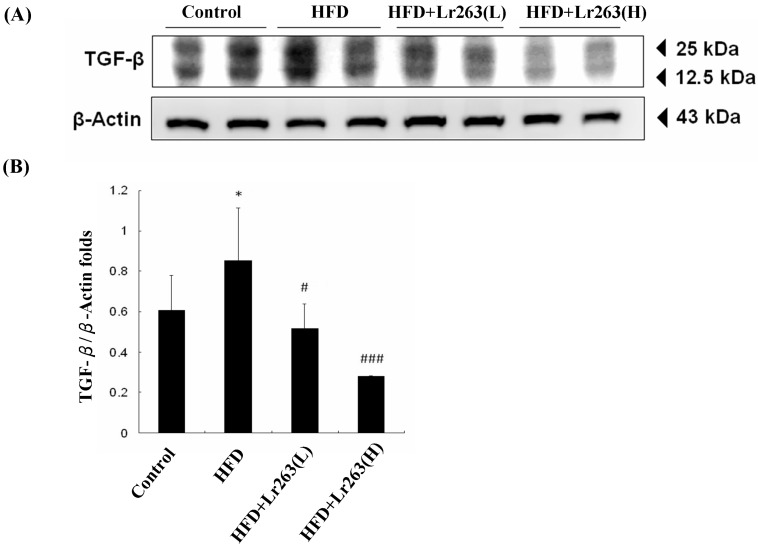
TGF-β protein levels in animal livers. (**A**) High TGF-β expression was observed only in the HFD group hamster livers; (**B**) The normalized protein expression of TGF-β with β-actin (* *p* < 0.05 compared with the control group, # *p* < 0.05, and ### *p* < 0.001 compared with the HFD group).

In this work, an autopsy of the HFD-induced fatty livers of the animals was used to evaluate fibrosis using Masson’s trichrome staining. For the HFD-only group hamster livers, the adipose capillaries that formed around the vacuoles are shown in Figure 2. However, collagen did not accumulate and cause fibrosis in the HFD-only group hamster livers. Furthermore, fatty liver disease and fibrosis were prevented in the heat-killed Lr263 (625 and 3125 mg/kg-hamster/day) treatment groups (Figure 2). The TGF-β protein level was higher in the HFD-only group than in the other hamster livers (Figure 3). Heat-killed Lr263 (625 mg/kg-hamster/day) treatment decreased TGF-β protein levels in the liver to a level similar to that of the control group, and heat-killed Lr263 (3125 mg/kg-hamster/day) decreased TGF-β protein expression to a level lower than that in the HFD-only hamster livers.

Interestingly, a possible mechanism underlying the effects of the probiotic is the interruption of cholesterol metabolism to replace the processed bile salts in the gut [24,26]. In this work, heat-killed Lr263 treatments also exerted a similar effect in the HFD hamsters. The lipid profiles of the livers and fecal analyses show a break-down in lipid absorption in the HFD hamsters after the heat-killed Lr263 treatments, as shown in Table 1. This result suggests a dose-dependent relationship between the heat-killed Lr263 treatments and lipid-elimination effects. Furthermore, the liver section results show higher TGF-β expression in the HFD hamster fatty liver (Figure 3), whereas the HFD hamster fatty liver only exhibits slight fibrosis (Figure 2).

Another *in vitro* study evaluated probiotic cholesterol assimilation in culture media and under simulated intestinal conditions; the results show that most *Lactobacillus* strains exhibit strong cholesterol assimilation and that *Lactobacillus reuteri* NCIMB 701089 assimilated over 67% of the cholesterol [39]. This result is similar to our results and suggests that the cholesterol assimilation ability of a probiotic is independent of whether they are alive or in a probiotic bacterial culture. The LPS and CpG DNA potentially possess the predominant bioactivities of a bacterium even after heat attenuation. Several TLRs (A Toll-like receptor recognizes bacterial DNA) were known to be induced by some bacterial LPS and CpG DNA. [40]. Reports show that certain *Lactobacillus* strains can evoke immunostimulatory effects through Toll-like receptor 2 (TLR-2); TLR-2 and TLR-4 are the key mediators of the inflammatory reaction in human visceral adipose tissue [41,42]. The relationship between heat-killed and living Lr263 cardiac protective effects, as well as those of TLRs, requires more experimentation.

**Table 1:** The body weight, food intake, lipid profile of hamster livers and fecal analyses.

Treatments	Control	HFD	HFD + Lr-263(L)	HFD + Lr-263(H)
**Parameter**				
Body Weight (g)	126.5 ± 10.6 *	138.3 ± 5.9	136.7 ± 3.8	124.7 ± 4.0 *
Food Intake (g)	8.1 ± 0.8	8.2 ± 0.9	7.9 ± 1.1	7.8 ± 0.5
**Liver**				
TG (mg/g)	85.0 ± 6.5 *	93.3 ± 5.3	90.7 ± 3.4	84.0 ± 4.3 *
T-CHO (mg/g)	89.7 ± 0.4 *	130.0 ± 4.9	118.3 ± 12.8	127.3 ± 6.0
MDA (μg/g)	4.2 ± 0.4 *	8.6 ± 0.7	6.7 ± 1.1	5.5 ± 0.4 *
**Fecal**				
TG (mg/g)	5.9 ± 0.7	4.7 ± 0.8	7.6 ± 0.9 *	10.4 ± 1.7 *
T-CHO (mg/g)	6.7 ± 0.7	8.5 ± 1.4 *	8.5 ± 1.4	11.0 ± 0.8 *

TG: triglyceride; T-CHO: total cholesterol; * *p*-value < 0.001 compared with the HFD group.

**Figure 4:**
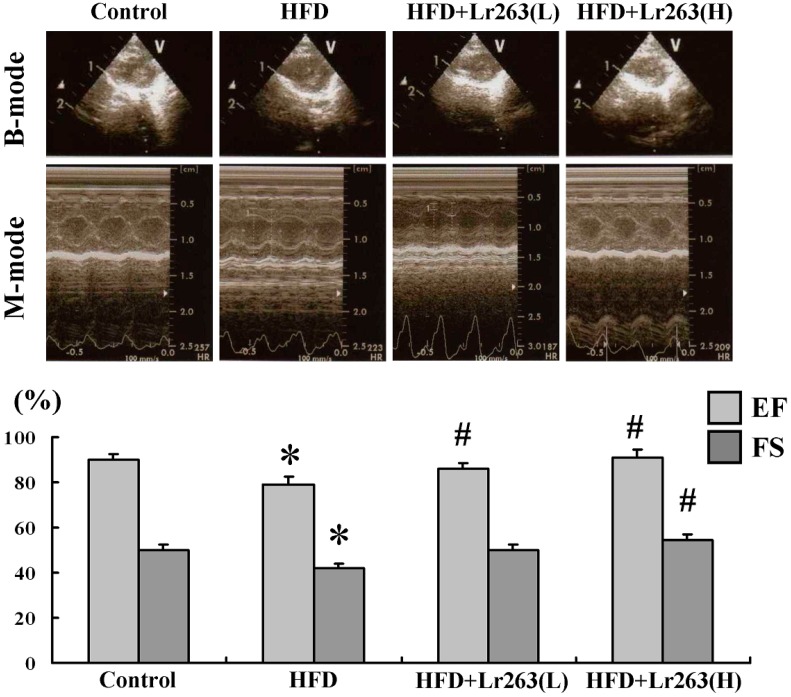
The echocardiography analysis of the hamster was performed using a 10 MHz transducer (GE 10S-RS). The B-mode was visualized for two-dimensional (long-axis and short-axis of the left ventricle) mode images and B-mode perspectives were further used to evaluate the left ventricle for the M-mode cursor. An M-mode evaluation of heart function was performed by comparing the left ventricular systolic and diastolic distances, which are shown as ejection fraction (EF) values and fractional shortening (FS) values (*n* = 6 in each group, * *p* < 0.05 compared with control group, and # *p* < 0.05 compared with HFD group).

In the echocardiographic analysis results, the heart function evaluated in the control group hamsters using fractional shortening (FS) was 55.10%; the ejection fraction (EF) was 90.09%; the FS was 42.11%, and the EF was 79.02% in the HFD-only hamster group (Figure 4). After heat-killed Lr263 (625 mg/kg-hamster/day) treatment, the FS improved to 50.00%, and the EF improved to 86.22%. Moreover, after heat killed Lr263 (3125 mg/kg-hamster/day) treatment, the FS improved to 56.52%, and the EF improved to 91.03%. Based on our previous work, TGF-β expression in the liver may regulate cirrhosis cardiomyopathy (CCM) in the early stage of CCl_4_-induced liver fibrosis [12]. Furthermore, a previous report showed that early treatment with a neutralizing anti-TGF-β antibody increased mortality in an infarcted heart [43]. However, experimental evidence shows that late TGF-β inhibition decreases collagen deposition and suppresses the number of myofibroblasts in wound remodeling after the inflammatory phase in an infarcted heart [44]. In this work, TGF-β expression in the HFD hamster heart may result in remodeling and affect HFD hamster heart function (Figure 4).

**Figure 5:**
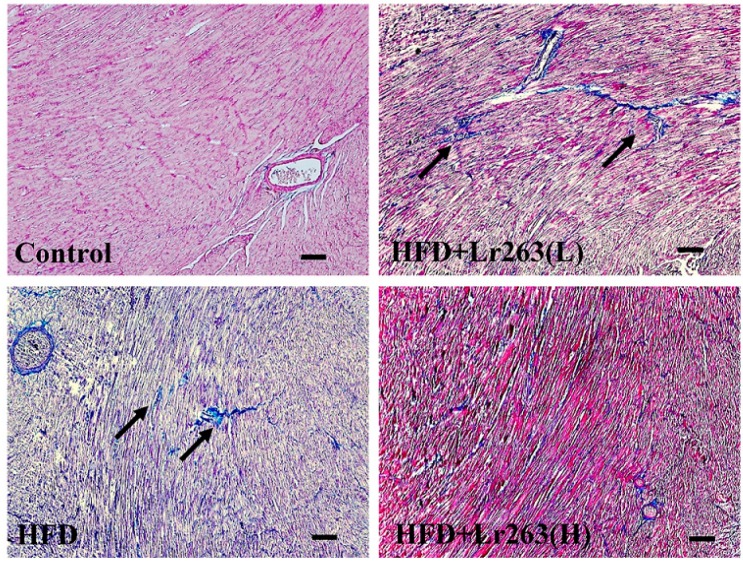
Assessment of fibrosis in the animal hearts. Cirrhosis was assessed using Masson’s trichrome (MS) staining assay to indicate collagen accumulation (the blue color indicated by the arrows) in the heart tissue slides. The bar length is 100 μm.

**Figure 6:**
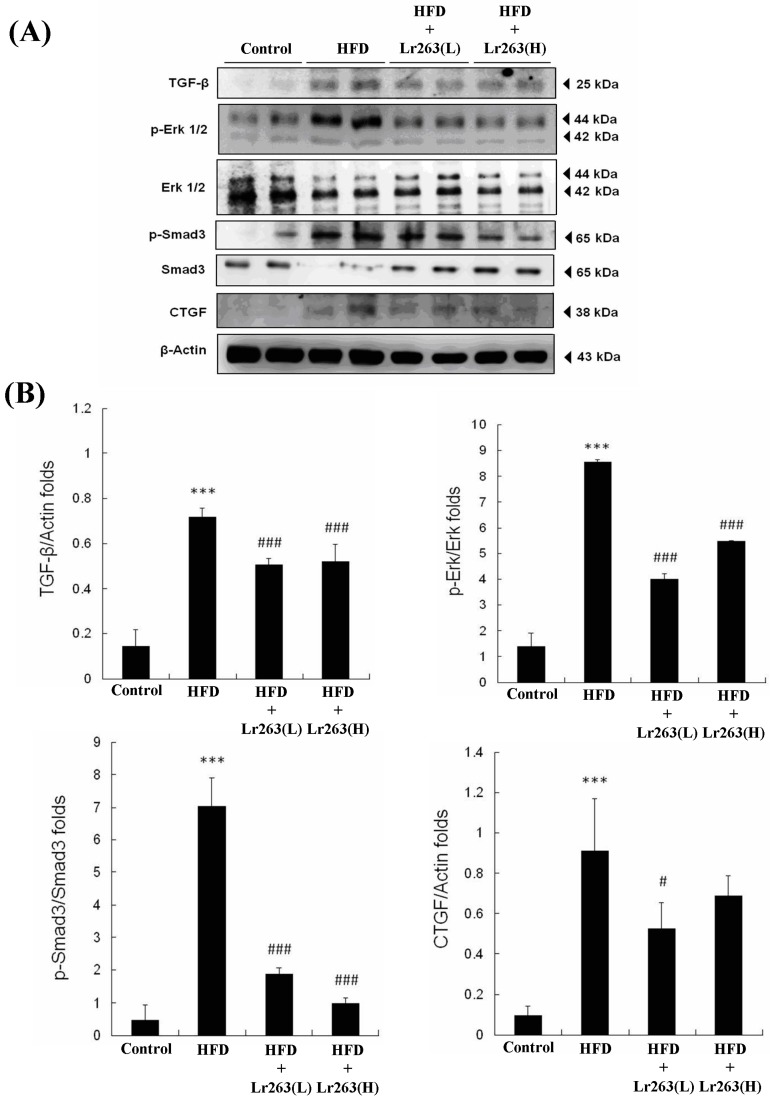
TGF-β pathway protein expression analysis. (**A**) The TGF-β/Smad-3/CTGF expression levels increased in the HFD treatment hamster hearts and decreased in the heat-killed Lr263 (625 and 3125 mg/kg-hamster/day) treatment hamster hearts; (**B**) The normalized protein expression of TGF-β/β-actin, p-Erk/Erk, p-Smad3/Smad3, and CTGF/β-actin (*** *p* < 0.001 compared with the control group, # *p* < 0.05, and ### *p* < 0.001 compared with the HFD group).

The sections with MT staining show fibrosis in portions of the HFD-only treatment hamster hearts (Figure 5). After heat-killed Lr263 (625 mg/kg-hamster/day) treatment, the fibrotic portions of the HFD treatment hamster hearts improved. Moreover, after heat-killed Lr263 (3125 mg/kg-hamster/day) treatment, the fibrotic portions in the HFD-only treatment hamster hearts exhibited more improvement. Furthermore, the TGF-β-induced fibrosis pathway was analyzed, and the TGF-β and its downstream proteins such as p-Erk, p-Smad3, and CTFG were highly expressed in the HFD-only treatment hearts (Figure 6). After heat-killed Lr263 (625 mg/kg-hamster/day) treatment, the TGF-β protein expression and downstream p-Erk, p-Smad3, and CTFG expression levels decreased in the HFD-only treatment hamster hearts.

Furthermore, Bujak *et al.* used mice with targeted disruption of Smad3, which showed no defect in inflammation resolution, but exhibited less fibrosis in the infarct; this result suggests that the Smad3-mediated fibrogenic actions of TGF-β do not regulate TGF-β anti-inflammatory functions [45]. The autopsy results show that TGF-β-induced fibrosis in certain portions of the HFD hamster hearts, which was slightly decreased by the heat-killed Lr263 (625 mg/kg-hamster/day) treatments (Figure 5). Furthermore, after heat killed Lr263 (3125 mg/kg-hamster/day) treatment, the TGF-β, p-Erk, p-Smad3, and CTFG protein levels in the HFD treatment hamster hearts decreased to levels similar to those of the control group. Only high-dose heat-killed Lr263 (3125 mg/kg-hamster/day) treatments reduced the TGF-β protein level and the expression levels of its downstream proteins; the data showed improvements in fibrosis induction in the HFD hamster heart (Figure 6).

## 3. Experimental Section

### 3.1. Preparing the Probiotic Suspensions

The Lr263 was deposited in the Bioresource Collection and Research Center, Taiwan (BCRC 910452) and China Center for Type Culture Collection, China (CCTCC M209263). The heat-killed Lr263 was provided by GenMont Biotech Inc., Tainan, Taiwan. The heat-killed Lr263 powder (8.0 × 10^8^ cells/g) was prepared from autoclaved Lr263. Next, it was diluted to the indicated probiotic concentrations (5 × 10^8^ and 2.5 × 10^9^ cells/mL) in the samples prepared for animal administration.

### 3.2. Animals

The protocol of animal use experimental in this work was approved by the Institutional Animal Care and Use Committee (IACUC) of China Medical University (No.101-263-B). All 24 male SD hamsters (eight weeks old, 300 g weight) were purchased from BioLASCO Taiwan Co., Ltd. (Taipei, Taiwan) and divided into four groups (*n* = 6 each). The control hamster group was labeled as control. The high-fat diet treatment hamster group was labeled as HFD. The hamster group with a high-fat diet combined with a low dose of heat-killed Lr263 (625 mg/kg-hamster/day) was labeled as HFD + Lr263(L). The hamster group with a high-fat diet combined with high dose of heat-killed Lr263 (3125 mg/kg-hamster/day) was labeled as HFD + Lr263(H). Oral administration of the probiotics was performed by gavage using a feeding needle, and the experiments were applied for eight weeks. All high-fat diet treatment hamsters were fed normal water *ad libitum* and a high-fat diet (HFD); the components of the normal diet and high-fat diet are listed in Table 2.

**Table 2:** Components of the normal diet and high-fat diet.

Components (g/kg)	Normal Diet	High-Fat Diet
Casein	200	232
l-Cystine	3.0	3.0
d,l-Methionine	N.D.	3.5
Corn Starch	397.48	137
Maltodextrin	132	150
Sucrose	100	162.58
Cellulose	50	50
Cholesterol	N.D.	1.9
Mineral Mix (AIN-93)	35	40.60
Calcium phosphate dibasic	N.D.	4.64
Vitamin Mix (AIN-93)	10	16.24
Choline Bitartrate	2.5	5
Tert-butylhydroquinone	0.014	0.04
Soybean oil	70	40
Lard	N.D.	153.5

N.D.: None detectable.

### 3.3. Cardiac Echocardiography

The small animal M-mode echocardiography analyses were performed using the Vivid I Ultrasound System (GE Healthcare, Milwaukee, WI, USA) via the parasternal long-axis and short-axis approach. B-mode previewing offers optimal positioning for the left ventricle (LV). M-mode measurements were performed from the B-mode perspectives and immediately recorded the left ventricular internal end-diastolic dimensions (LVIDd) and left ventricular internal end-systolic dimensions (LVIDs). Fractional shortening (FS%) was presented as the calculated results using the following equation: [(LVIDd − LVIDs)/LVIDd] × 100. The ejection fraction (EF) means the percentage of the blood volume pumped out from LV.

### 3.4. Masson’s Trichrome Staining

The 2 μm thick paraffin sections of each group hamster hearts and livers were cut from paraffin-embedded tissue blocks. The slices were deparaffinized and rehydrated before further staining. The samples were then subjected to with Masson’s trichrome staining and investigate the histological and fibrotic changes in heart and liver sections. The photomicrographs were obtained using microscopes (Zeiss Axiophot, Oberkochen, Deutschland, Germany) under the 200× magnification.

### 3.5. RNA Extraction and RT-PCR

The total RNA for all liver tissues was isolated using Trizol single-step RNA isolation reagent (Invitrogen Life Technologies, BRL, Carlsbad, CA, USA) and then subjected to reverse transcription using an RT-PCR (Promega, San Luis Obispo, CA, USA). The PCR primers used for fatty acid synthase (FAS) were as follows: forward, 5′-GTGGAAGGCTGGGCTCTATG-3′; and reverse, 5′-AGGCGTCGAACTTGGACAGA-3′. The primers used for peroxisome prolifera proliferator-activated receptor γ (PPARγ) were as follows: forward, 5′-TCAGGTTTGGGCGAATGC-3′; and reverse, 5′-GGGTTCAGCTGGTCGATATCAC-3′. The primers used for 3-hydroxy-3-methylglutaryl-coenzyme A reductase (HMG-CoA reductase) were as follows: forward, 5′-TGTGGGAACGGTGACACTTA-3′; and reverse, 5′-CTTCAAATTTTGGGCACTCA-3′. The primers used for LDL-cholesterol receptor (LDLR) were as follows: forward, 5′-AGCCGATGCATTCCTGACTC-3′; and reverse, 5′-AGTTCATCCGAGCCATTTTCAC-3′. The primers used for cholesterol 7α-hydroxylase (CYP7A1) were as follows: forward, 5′-ACGTGGTTGGAAGAAGCG-3′; and reverse, 5′-GAATGTGGGCAGCGAGAA-3′. The primers used for β-actin were as follows: forward, 5′-AGGGAAATCGTGCGTGACA-3′; and reverse, 5′-GTGGCCATCTCTTGCTCGAA-3′. The reaction mixtures were maintained at 48 °C for 45 min and then heated to 94 °C for 2 min for reverse transcription using the following protocol: 94 °C for 30 s of denaturation, 60 °C for 1 min of annealing, 68 °C for a 2 min extension for 40 cycles, and one cycle of 7 min for a final extension in a PerkinElmer PCR machine. Each PCR product result was normalized and expressed as the relative density to the β-actin gene. We also tested GAPDH as a reference gene and it gave similar results to β-actin.

### 3.6. Tissue Protein Extraction

Heart tissue protein samples were extracted and homogenized from six hamsters in each group using the lysis buffer contents 0.05 M Tris-HCl, 0.15 M NaCl, 0.25% deoxycholic acid, 1% nonyl phenoxypolyethoxylethanol, and 1 mM EDTA at pH = 7.4. The supernatants were collected form the homogenates after centrifuged at 13,000 rpm for 40 min. The protein concentration of each sample was calibrated and then stored the samples at −80 °C for further experiments.

### 3.7. Western Blot Assay

Heart tissue proteins were separated in a 12% SDS polyacrylamide gel electrophoresis (SDS-PAGE) and transferred to a Hybond-C membrane (GE Healthcare UK Ltd., Little Chalfont, Buckinghamshire, UK). Then 3% bovine serum albumin (BSA) in a tricine buffer solution was used to block the Hybond-C membrane. After 30 min BSA blocking and additional three times PBS washing, the primary antibodies were added to identify the indicated proteins. The primaries used in this work were including β-actin (sc-47778, Santa Cruz Biotechnology, Dallas, TX, USA), CTGF (sc-1745, Santa Cruz Biotechnology), p-Erk (sc-7382, Santa Cruz Biotechnology), Erk (sc-94, Santa Cruz Biotechnology), and TGF-β (sc-31609, Santa Cruz Biotechnology). After the antibodies’ reorganizations, horseradish peroxidase-labeled antibodies were used and pictures were then taken using a Fujifilm LAS-4000 (GE Healthcare UK Ltd.).

### 3.8. Liver and Fecal Lipid Profile Analysis

All of the liver and fecal samples were freshly collected after the experiments, and 100 mg of each sample was lyophilized, weighed, and then homogenized in 5 ml of chloroform-methanol (*v*/*v* = 2:1) solution. All of the solution samples were further analyzed using a triglyceride quantification assay kit (ab65336, Abcam, Taipei, Taiwan) and cholesterol/cholesteryl ester quantitation assay kit (ab65359, Abcam) in accordance with the protocols suggested by the manual.

### 3.9. Statistical Analysis

The results were obtained from six hamsters of each experimental group and are represented as the group mean ± standard deviation (SD). One-way analysis of variance was used to indicate an overall statistical significance among the means of the four experimental groups. A p-value less than 0.05 was considered significant. Statistical analyses were performed using SigmaPlot v.10.0 software (San Jose, CA, USA).

## 4. Conclusions

In conclusion our current findings show that Lr263 daily oral gavage treatment may reduce the lipid metabolic stress in liver, and attenuate the cardiac fibrosis by suppressing the TGF-β expression. Our results suggest that heat-killed Lr263 supplementation would potentially improve the health of the liver and heart.
